# Rapidly Fatal Hemorrhagic Pneumonia and Group A *Streptococcus* Serotype M1

**DOI:** 10.3201/eid2001.130233

**Published:** 2014-01

**Authors:** Maria Santagati, Teresa Spanu, Marina Scillato, Rosaria Santangelo, Fabio Cavallaro, Vincenzo Arena, Giacomo Castiglione, Marco Falcone, Mario Venditti, Stefania Stefani

**Affiliations:** University of Catania, Catania, Italy (M. Santagati, M. Scillato, S. Stefani);; Catholic University of the Sacred Heart, Rome, Italy (T. Spanu, R. Santangelo, F. Cavallaro, V. Arena);; Vittorio Emanuele Hospital, Catania (G. Castiglione);; University of Rome La Sapienza, Rome (M. Falcone, M. Venditti)

**Keywords:** Hemorrhagic pneumonia, group A Streptococcus, GAS, streptococci, bacteria, serotype M1

## Abstract

We report 3 cases of fulminant hemorrhagic pneumonia in previously health patients. Sudden-onset hemoptysis and dyspnea developed; all 3 patients and died <12 h later of massive pulmonary bleeding, despite aggressive supportive care. Postmortem analysis showed that the illnesses were caused by group A *Streptococcus* *emm1*/sequence type 28 strains.

*Streptococcus pyogenes* or group A *Streptococcus* (GAS) is a versatile pathogen that can cause serious diseases, including bacteremia, cellulitis, puerperal sepsis, meningitis, pneumonia, and necrotizing fasciitis (*1**,**2*). This pathogen also causes streptococcal toxic shock syndrome, a severe response to streptococcal pyrogenic exotoxins (Spe proteins), which trigger overproduction of inflammatory cytokines, leading to tissue damage, organ failure, and shock (*3**,**4*). Despite improved awareness and treatment, GAS infections remain among the top 10 infectious causes of fatal disease in humans (*1*).

Periodic resurgences of invasive GAS infections in industrialized countries have been linked to emergence of dominant GAS clones resulting from horizontal gene transfer (*5**–**8**).* The ability of these clones to cause life-threatening disease in healthy persons probably depends on expression of virulence factors that facilitate penetration of host cell barriers and evasion of immune defenses (*3**,**4**,**8*).

More than 250 GAS types have been identified in sequencing studies of the hypervariable region of the *emm* gene, which encodes the M surface protein (*1**,**2*). The *emm* types seem to correlate with tissue tropism of the organism, and M protein augments GAS virulence by interfering with antibody and complement deposition, facilitating formation of microcolonies and neutralizing antimicrobial peptides, and stimulating proinflammatory and procoagulatory activities (*3**,**4*). M1 protein and M1 fragments released by neutrophil proteases can also provoke pulmonary hemorrhage, inflammation, and tissue destruction by their interaction with fibrinogen, which causes neutrophils to release heparin-binding protein, a mediator of vascular leakage (*9*). The hypervirulent *emm*1 GAS clone also harbors prophages encoding SpeA proteins and extracellular streptodornase D (Sda1), which can also enhance virulence and dissemination (*8**,**10**).* We report 3 cases of rapidly fatal, hemorrhagic pneumonia in previously healthy patients that were determined to be caused by infection with hypervirulent GAS.

## The Study

Patient 1 was a 74-year-old Italian man who came to the emergency department (ED) of Vittorio Emanuele Hospital in Catania, Italy, in July 2012. Patient 2 was 65-year-old German woman who arrived in Rome from Frankfurt in March 2011 and came directly from the airport to the ED of the University of Rome Medical Center. Patient 3 was a 40-year-old Japanese man who came to the ED of the Catholic University Medical Center in Rome in February 2012, 3 days after arriving in the city. All 3 patients had sudden-onset hemoptysis, dyspnea, and fever (temperature 37.5–38.5°C), which had rapidly worsened over 2–3 h. Their medical histories were unremarkable.

All 3 patients had acute respiratory failure requiring ventilator support and hemoconcentration; hyponatremia; increased levels of serum creatinine, lactate dehydrogenase, lactic acid, and brain natriuretic peptides; leukocytosis (7,000–8,000 cells/mm^3^, 3,200–4,000 neutrophils); and increased levels of C-reactive protein (>400 mg/mL) and d-dimer (>2,000 μg/mL). Computed tomography of the chest showed patchy opacification throughout the lungs and multifocal confluent parenchymal opacities (Figure, panel A). Bronchoalveolar lavages were bright red and contained numerous erythrocytes, gram-positive cocci resembling streptococci, and no polymorphonuclear leukocytes. In spite of aggressive supportive care and empirical therapy with ceftriaxone (2 g intravenously) and levofloxacin (500 mg intravenously), the conditions of the patients deteriorated rapidly, and all 3 died of massive pulmonary hemorrhage ≤12 h after symptom onset.

**Figure:**
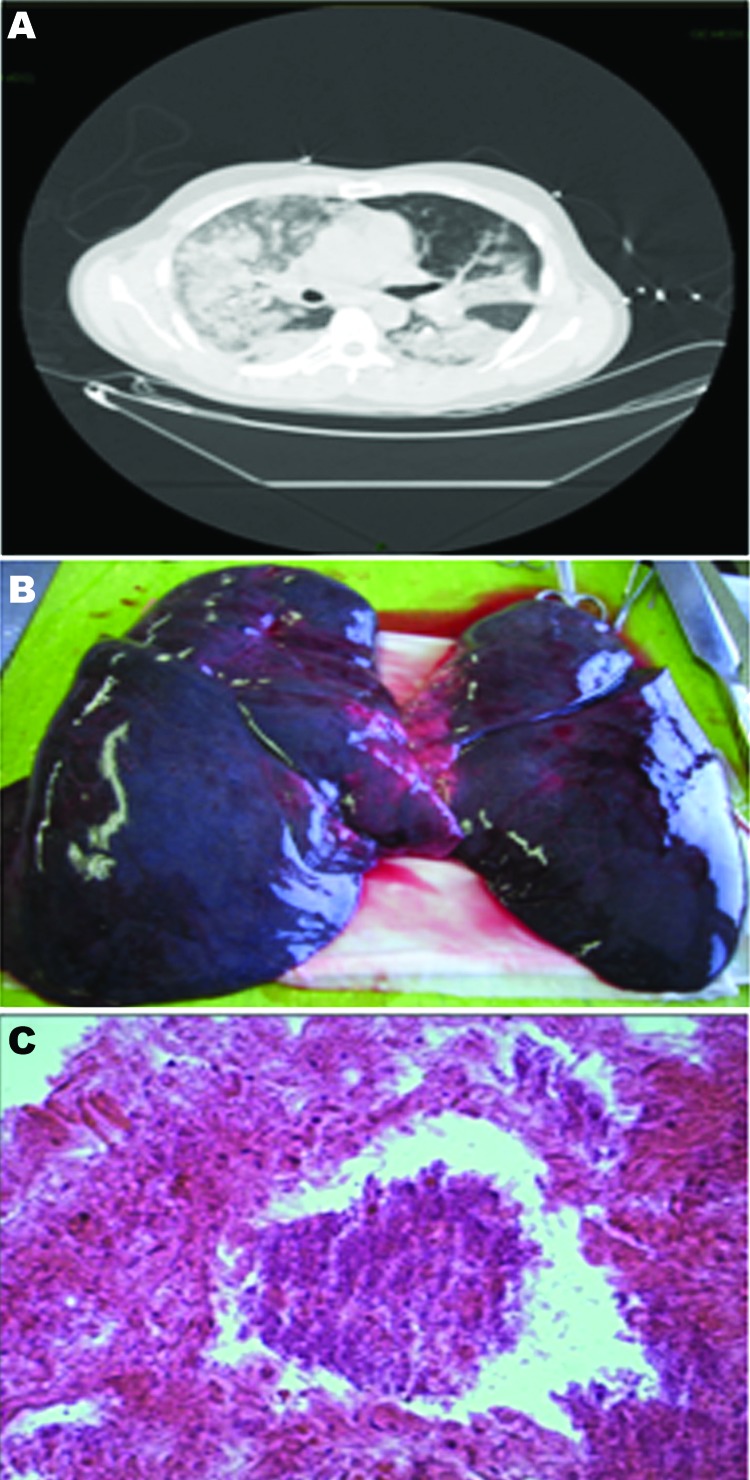
A) Computed tomographic image of the chest of a 74-year-old patient (patient 1) with fatal hemorrhagic pneumonia, Catania, Italy, showing multifocal confluent parenchymal opacities. B) Postmortem view of the lungs showing hemorrhage and edema. C) Microscopic evidence of necrosis and bacteria in the lungs (original magnification ×40).

Autopsy specimens showed bilateral hemorrhagic pleural effusions (1,500–2,000 cells/mL), heavy, blood-engorged lungs (1,600–1,700 g), and patent hilar structures, but no thoracic or abdominal lymphadenopathy. Lungs showed hemorrhage and edema (Figure, panel B). Microscopic analysis of the lungs showed necrosis and bacteria (Figure, panel C). Admission blood cultures (3 sets/patient) were positive at the 12-h reading. *S. pyogenes* was identified by using matrix-assisted laser desorption ionization–time-of-flight mass spectrometry (Bruker Daltonik, Breman, Germany) and isolated in all culture bottles, bronchoalveolar lavage cultures (pure colonies, 10^6^ CFU/mL), and postmortem lung tissue cultures.

All isolates were susceptible to erythromycin, tetracycline, amoxicillin, penicillin, and clindamycin by Etest (bioMérieux, Marcy l’Etoile, France). Results were interpreted according to European Committee on Antimicrobial Susceptibility Testing breakpoints (www.eucast.org/clinical_breakpoints). Results of testing for urinary *Legionella pneumophila* and *S. pneumoniae* antigens, β-glucan, galactomannan, and HIV and toxicology panels were negative for all 3 patients. Commercially available PCRs for respiratory tract samples showed negative results for major respiratory viruses and bacterial pathogens. Antibody titers against streptolysin O were <200 IU.

The *emm* typing, which was performed by PCR using protocols and the database of the Centers for Disease Control and Prevention (Atlanta, GA, USA; www.cdc.gov/ncidod/biotech/strep/M-ProteinGene_typing.htm), showed that all 3 GAS strains were *emm*-type 1 and had identical sequences at the 5ʹ end of the *emm* gene, indicative of the *emm* 1.0 allele. Strains were tested for multiple virulence genes (Table) by using PCR and primers described elsewhere (*11*) or designed with the VectorNTI program (Invitrogen, Carlsbad, CA, USA). They were identical in terms of the *spe* genotype (*speA*+, *speB*+, *speC–*, *speG*+, *speI–*, *speJ*+, *smeZ*+, *ssa*–*)*; the presence of *SLO*, *sag*A, *sag*BC, and *sda*1 genes; and the absence of *PAM*, *prt*F, and *sof* genes. Multilocus sequence typing was performed as described (http://spyogenes.mlst.net/) and showed an identical sequence type (ST) (ST28/CC28) for the isolates from patients 1 and 3 and a new single-locus variant of ST28 (designated ST648) for the isolate from patient 2. All cases were caused by GAS strains harboring the *emm1.0* allele and *Sda1*, *smeZ, speA, speB, speG*, and *speJ* genes. 

**Table:** Primers used for identification of virulence genes by PCR for group C *Streptococcus* isolates*

Gene	Primer name†	Primer sequence, 5′→3′	Amplicon size, bp
*Slo*	MS442	GGTAACCTTGTTACTGCTAATGCTGA	400
	MS443	TAATGGAAATATCGACTGGTGTAGTG‡	400
*speA*	speA-fw	CTCAACAAGACCCCGATCCAAG	500
	speA-rew	ATTTAGAAGGTCCATTAGTATATAGTTGC‡	500
*speB*	MS384	GGCATGTCCGCCTACTTTACCGA	800
	MS385	CAGGTGCACGAAGCGCAGAAG‡	800
*speC*	MS410	TACTGATTTCTACTATTTCACCTATCATC	447
	MS411	TCTGATTTTAAAGTCAATTTCCTGG‡	447
*speG*	MS412	GCTATGGAAGTCAATTAGCTTATGCAG	448
	MS413	CCGATGTATAACGCGATTCCGA‡	448
*speI*	speI-up	GGTCCGCCATTTTCAGGTAGTTT	516
	speI-rew	ACGCATACGAAATCATACCAGTAG‡	516
*speJ*	MS414	CACTCCTTGTACTAGATGAGGTTGC	508
	MS415	ACGCATACGAAATCATACCAGTAG‡	508
*sagBC*	sagBC-fw	GCAGCTAGTTGCTCAACATTTAATG	600
*sagBC*	sagBC-rew	CATAGGCAGTCGCCTGATTCC‡	600
*prtF*	MS400	CGGAGTATCAGTAGGACATGCGGA	882
*prtF*	MS401	CTCCCACCAACATTGCTTAATCCA‡	882
*PAM*	PAM-fw	GCAGACGACGCTAGAAATGAAGTA	900
	PAM-rew	CCTGCTTGTGGTGCTTGACCTTTAC‡	900
*Sof*	MS402	ATGCCTGGTTGGGTATCTTCGGT	406
	MS403	AGAGAACAAAACGTTCTGCGCCTA‡	406
*Ssa*	ssa-fw	GTAGTCAGCCTGACCCTACTCCAGAAC	621
	ssa-rew	ACTGATCAAATATTGCTGCAGGTGC‡	621
*sda1*	MS431	GGGTCTATAAGAAAAGTGGGCAAAG	439
	MS432	TGATCGTAAAGGTGGGATGCAGTA‡	439

*slo, streptolysin O; *sag*A/BC, streptolysin S-associated gene A protein; *smeZ-2* mitogenic exotoxin Z; *speA*, streptococcal pyrogenic toxin A; *speB*, streptococcal cysteine protease; *speC*, streptococcal pyrogenic toxin C; *speG,* streptococcal pyrogenic toxin G; *speI*, streptococcal pyrogenic toxin I; *speJ;* streptococcal pyrogenic toxin J; *prtF,* fibronectin-binding protein; *PAM*, plasminogen-binding protein; *sof* serum opacity factor; *ssa* streptococcal superantigen; *sda*1, streptodornase D. †Primers indicated in **boldface** were created with the VectorNT program (Invitrogen, Carlsbad, CA, USA). Other primers were described by Santagati et al. (*11*). ‡Reverse primers.

## Conclusions

A total of 1.3%–23.8% invasive GAS infections involve pneumonia, but these infections are more common in developing countries (*1**,**6**,**7**,**12*). Successful management depends on early diagnosis and initiation of effective treatment (*2**,**3*). However, despite early aggressive supportive care and empirical antimicrobial drug therapy, which was later confirmed to be appropriate by antibiogram results, all 3 patients we describe died within 8–12 h of symptom onset from massive pulmonary bleeding and acute respiratory failure. Initial presentations were fairly nonspecific, and diagnoses were made postmortem. The roles of GAS in the rapid progression of disease were highlighted by the abundance of GAS found the lungs, extensive and severe pulmonary damage, and virulence factor profiles of the isolates, all of which included *sda1*, *smeZ*, *speA*, *speB*, *speG*, *speJ*, and *emm-1* genes (*4**,**5*).

Invasive bacterial disease requires virulence factors that facilitate interactions of the microbe with host tissues and subvert defenses of the immune system. In the *emm1* GAS clone, progression to systemic infection is also favored by mutations in the 2-component control of virulence regulatory system, which enhances resistance to subepithelial immune defenses and facilitates deep-tissue penetration. These mutations markedly alter transcription profiles of invasive GAS isolates than those of pharyngeal mucosal isolates (*3**–**5**,**8*), strongly upregulating *sda1* transcription and markedly downregulating expression of the gene encoding cysteine protease SpeB (*6**–**12*). The *sda1* gene facilitates avoidance of neutrophil extracellular traps by the pathogen and serves as a selective force for a control of virulence regulatory system mutation (*7**,**13*). SpeB protease enables accumulation and activation of broad-spectrum host protease plasmin on the microbial cell surface, thereby promoting infection spread to normally sterile sites (*2**,**3**,**14*). Invasive GAS strains also produce increased levels of toxins, including some that destroy immune cells, and superantigens (e.g., SpeA, SpeJ) that dysregulate the immune response of the host (*3*).

Host factors also affect clinical presentation and disease progression, which explains why diseases of different severity can be caused by genetically indistinguishable *emm1* strains with no evidence of regulatory gene mutation (*2**,**4**,**7*). Elucidation of these host and bacterial factors involved in the pathogenesis of these rare but life-threatening infections may be useful for improving disease prognosis (*6*).
